# Screening and Identification of Garlic Leaf Blight (*Pleospora herbarum*)-Resistant Mutants Induced by Ethyl Methane Sulphonate

**DOI:** 10.3390/ijms241411819

**Published:** 2023-07-23

**Authors:** Yuanyuan Hong, Yinhui Sun, Xuan Zhang, Lingling Zhang, Xin Yuan, Zhaoyang Ma, Meiqian Wu, Shuxia Chen

**Affiliations:** Shaanxi Engineering Research Center for Vegetables, College of Horticulture, Northwest A&F University, Yangling, Xianyang 712100, China; yuanyuanhong@nwsuaf.edu.cn (Y.H.); 412725@nwsuaf.edu.cn (Y.S.); zhangxuan19980729@nwafu.edu.cn (X.Z.); zhanglingling1019@nwafu.edu.cn (L.Z.); yuanxin2020050359@nwafu.edu.cn (X.Y.); mzybetter@nwafu.edu.cn (Z.M.); wmqian@nwafu.edu.cn (M.W.)

**Keywords:** garlic, chemical mutagenesis, leaf blight, agronomic traits, directional selection

## Abstract

Garlic (*Allium sativum* L.) is a popular condiment used as both medicine and food. Garlic production in China is severely affected by continuous cropping and is especially affected by leaf blight disease. Garlic is sterile, so it is very important to develop specialized genotypes, such as those for disease resistance, nutritional quality, and plant architecture, through genetic modification and innovation. In this experiment, we applied the induction method using EMS to mutate garlic cloves of cultivar G024. From the mutations, 5000 M_0_ mutants were generated and planted in the field. Then, 199 M_1_ mutant lines were screened according to growth potential and resistance to leaf blight. From M_2_ to M_3_, 169 generational lines were selected that grew well and were resistant to leaf blight in the field. Thereafter, their resistance to leaf blight was further analyzed in the lab; 21 lines resistant to leaf blight that had good growth potential were identified, among which 3 mutants were significantly different, and these were further screened. Also, transcriptome analysis of two mutants infected with *Pleospora herbarum*, A150 and G024, was performed, and the results revealed 2026 and 4678 differentially expressed genes (DEGs), respectively. These DEGs were highly enriched in hormone signaling pathway, plant–pathogen interaction, and MAPK signaling pathway. Therefore, the results provide a theoretical and technical basis for the creation of garlic germplasm resistant to leaf blight.

## 1. Introduction

Garlic (*Allium sativum* L.) is a commonly produced vegetable due to its high demand by the food industry and consumers. The production of garlic in China is approximately 80% of the total production in the world, and it has only increased in recent years. It is an important, high-value crop mainly cultivated in the central and northwestern regions [1]. Continuous cropping of garlic to increase production and economic efficiency ultimately leads to the accumulation of different pathogens, such as leaf blight and rust [2]. Garlic is prone to leaf blight during the production process, which, in severe cases, can reduce production up to 70% [3]. Continuous cropping can cause the accumulation of pathogens such as the fungus *Stemphylium solani*, which results in outbreaks of leaf blight. When the leaves are infected by the fungus, white spots form and then enlarge into sunken lesions, extending until the leaves wither [4]. In recent years, leaf blight has become lethal to garlic, primarily attacking garlic leaves, stalks, and bulbs [5]. Since spores of *Stemphylium solani* can spread air, healthy plants are readily infected. Garlic propagates vegetatively and there is hardly any natural mutation, so it is critically important to develop specialized genotypes, such as disease resistance, through genetic modification and innovation.

Ethyl methane sulphonate (EMS) is an alkylating chemical mutagen that can cause high frequency gene mutations and low frequency chromosomal aberrations [6,7]. It is widely used in chemical mutagenesis and is currently recognized as a chemical mutagen with obvious and stable effects. At present, this mutagen has achieved satisfactory results in the breeding of sexually propagating materials; for example, wheat plants that are salt-tolerant [8], stem rust-resistant [9], and Fusarium-head-blight-resistant [10] can be obtained, and rice strains that are herbicide-resistant [11] and drought-tolerant [12] can be bred. In addition, resistant plants can be obtained by EMS mutagenesis in asexually propagating plants, such as drought-tolerant [13,14], herbicide-resistant [15], and smut-resistant [16] sugarcane strains; salt-tolerant strawberry plants [17]; and blight-resistant potato varieties [18]. This technology overcomes the breeding difficulties of asexually propagating materials, and at the same time provides a technical foundation for future generations to cultivate resistant materials. Garlic is an asexually propagating material, and it is difficult to cultivate leaf-blight-resistant plants through conventional breeding methods; EMS mutagenesis is thus an efficient and high-quality breeding method to obtain leaf-blight-resistant garlic plants.

Leaf blight is a fungal disease that commonly occurs on allium and garlic plants [3] and decreases garlic yield and quality. At present, leaf blight is mainly controlled by using chemical agents or breeding resistant varieties. Although some chemical agents can prevent and control the occurrence of leaf blight to a certain extent, large quantities of such agents will not only pollute the environment, but also lead to food safety risks [19], and chemical agents cannot fundamentally prevent the occurrence of leaf blight. Thus, the selection and breeding of disease-resistant plants can potentially minimize the occurrence of leaf blight. Due to low natural variability [20], it is difficult to obtain disease-resistant plants through conventional breeding methods. Therefore, it is necessary to develop efficient methods to remedy this defect and speed up the process of obtaining mutants. EMS mutagenesis can increase the mutation frequency of plants to obtain mutants with specific traits. In short, the use of EMS mutagenesis to obtain leaf-blight-resistant garlic strains is efficient, fast, and feasible.

The purpose of this experiment was to use EMS as a mutagen to construct a mutant library by direct mutagenesis of garlic cloves. Field observations and greenhouse inoculation were carried out to identify and screen out plants with stable heredity against leaf blight. This experiment may provide a theoretical and technical basis for the creation of new garlic germplasms resistant to leaf blight and for future research on garlic breeding.

## 2. Results

### 2.1. Selection of EMS-Induced Mutant with Garlic G024

**Figure 1:**
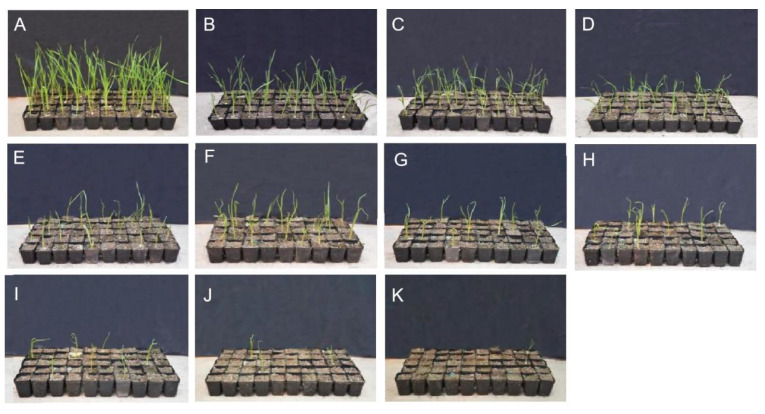
Garlic clove germination after treatment with EMS: (**A**) CK; (**B**) 1.0% EMS for 4 h; (**C**) 1.5% EMS for 4 h; (**D**) 1.0% EMS for 8 h; (**E**) 1.5% EMS for 8 h; (**F**) 1.0% EMS for 12 h; (**G**) 1.5% EMS for 12 h; (**H**) 1.0% EMS for 16 h; (**I**) 1.5% EMS for 16 h; (**J**) 1.0% EMS for 24 h; (**K**) 1.5% EMS for 24 h.

In this study, garlic variety G024 was used as test material to study whether EMS could induce mutations in garlic cloves. First, a chemical culture system suitable for G024 was established to screen mutants. We found that different concentrations of EMS and different times had different effects on the germination rate of garlic cloves. The overall trend was that with increasing EMS concentration and extended treatment time, the germination rate of garlic cloves, as well as the plant height, gradually decreased (Figure 1).

When the EMS concentration was 1.0% and treatment time was 8 h, the germination rate of garlic cloves decreased from 99.33 to 49.33%, which was significantly different from that of the control. When the concentration of EMS was increased to 1.5% and the treatment time remained unchanged, the germination rate decreased to 43.33%, and when the concentration of EMS was 1.0% and the treatment time was extended to 24 h, the germination rate decreased to 6.0% (Table 1), indicating that increasing the concentration of EMS and extending the treatment time will increase the physiological damage from EMS to garlic cloves and decrease the germination rate. After treatment with 1.0% EMS for 8 h, the germination rate of bulbils was 49.33%, which was close to the semi-mortality rate (50%). Therefore, it was determined that 1% EMS for 8 h was the best mutagenesis condition for garlic cloves.

**Figure 2:**
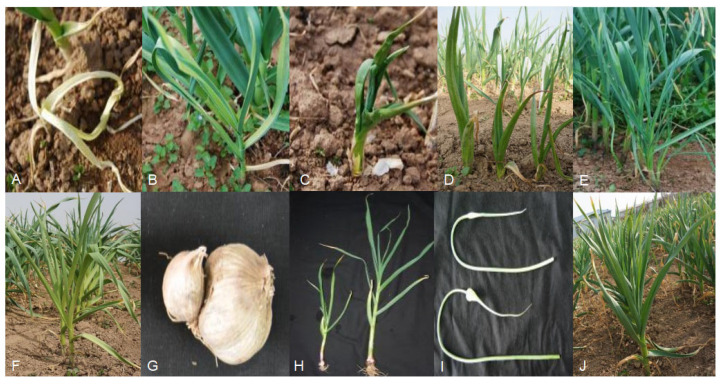
Morphological observations of mutants: (**A**) leaf albinism; (**B**) mosaicism; (**C**) curly leaves; (**D**) pseudo-stem disappearance; (**E**) abnormal tillering; (**F**) leaf yellowing; (**G**) secondary growth of bulbs; (**H**) plant dwarfing; (**I**) involucre enlargement; (**J**) G024.

The phenotypic traits of the M_1_ mutants were observed during the growth period. A total of 3176 lines were observed (the remaining garlic cloves did not germinate), and 199 lines showed variation, with a variation rate of about 6.2% of phenotypic traits (Table 2). Some obvious phenotypic traits in mutants were recorded, such as leaf albinism (Figure 2A), mosaicism (Figure 2B), curly leaves (Figure 2C), pseudo-stem disappearance (Figure 2D), abnormal tillering (Figure 2E), leaf yellowing (Figure 2F), secondary growth of bulbs (Figure 2G), plant dwarfing (Figure 2H), and involucre enlargement (Figure 2I).

### 2.2. Variation of Agronomic Traits in M_2_ Mutants

To observe the differences between agronomic traits of the mutants’ progeny, the LL, LW, LN, HP, SD, GW, GD, GH, and PN of M_2_ mutants were measured. Compared with the G024 cultivar, the main agronomic characteristics of the M_2_ generation mutants showed some changes. The coefficient of variation (CV) of M_2_ and G024 was calculated to understand the effective variation. Among the traits, PN, LL, SD, GW, and LW showed a higher degree of mutation, while HP and PN showed a lower degree (Table 3).

**Figure 3:**
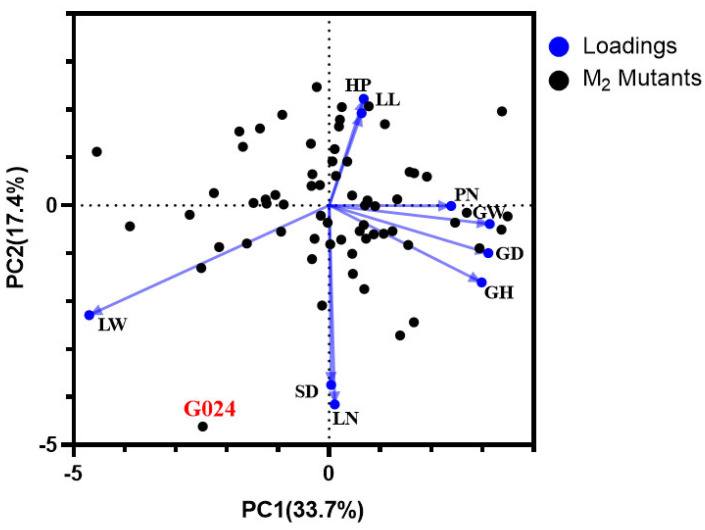
Comparison of nine agronomic traits between M_2_ mutants and G024. Distribution of agronomic traits plotted by principal component analysis (PCA). LL, leaf length; LW, leaf width; LN, leaf number; HP, pseudo-stem height; SD, stem diameter; GW, garlic weight; GD, garlic diameter; GH, garlic height; PN, petal number.

Furthermore, PCA was performed to comprehensively evaluate LL, LW, LN, HP, SD, GW, GD, GH, and PN between the M_2_ mutants and G024 cultivar in order to identify the agronomic traits responsible for the variable discrimination. The results showed that two principal components (PCs) together explained 51.1% of the variance in the whole dataset (PC1: 33.7%; PC2: 17.4%). The distribution of traits for the two components (PC1 and PC2) is shown in Figure 3. It can be observed that the mutagenic lines cluster together whereas the GO24 controls are outliers.

### 2.3. Variation of Agronomic Traits in M_3_ Mutants

Further analysis of the degree of variation for each M_3_ mutant trait showed that GW and LL had larger variations, while PN and LN had smaller variations (Table 4). The degree of variation for GW, LL, and SD was similar to that of M_2_ mutants, indicating that these three traits were no longer separate in the M_3_ generation.

**Figure 4:**
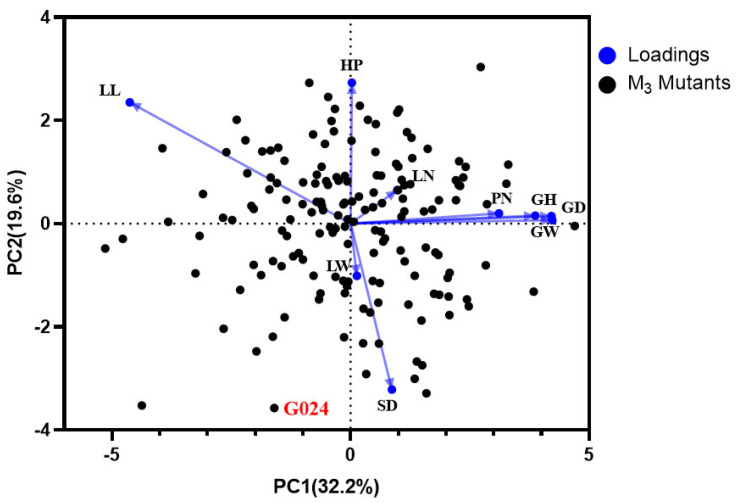
Comparison of nine agronomic traits between M_3_ mutants and G024. Distribution of agronomic traits plotted by principal component analysis (PCA). LL, leaf length; LW, leaf width; LN, leaf number; HP, pseudo-stem height; SD, stem diameter; GW, garlic weight; GD, garlic diameter; GH, garlic height; PN, petal number.

PCA of nine traits in the M_3_ mutants showed that two principal components (PCs) combined explained 51.8% of the variance in the entire dataset (PC1: 32.2%; PC2: 19.6%). It can be seen from Figure 4 that HP was the main contributor to PC1, and PN, GW, GD, and GH were the main contributors to PC2. The mutant lines were clustered together, while the G024 control group was an outlier, which is consistent with the M_2_ mutants. These results suggest that G024 has great plant growth and development advantages.

### 2.4. Screening and Identification of Mutants Resistant to Leaf Blight

**Figure 5:**
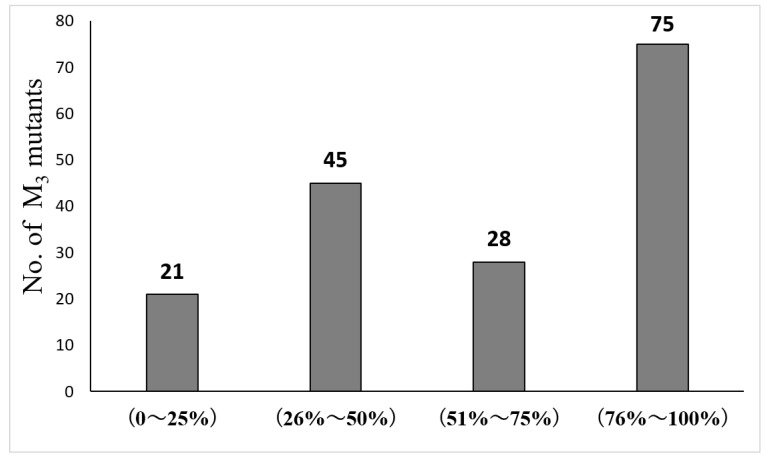
Division of selected mutant lines of garlic into four groups based on leaf blight index values. X-axis: group number and range of leaf blight index scores. Y-axis: number of lines in each group. Data label over each bar corresponds to number of lines in that group.

Garlic is susceptible to leaf blight, which can decrease production. In the summer of 2020, all M_3_ mutants were infected with leaf blight to varying degrees, and their disease index was measured. It can be seen from Figure 5 that the mutant lines had different levels of tolerance to leaf blight, and 21 mutant lines showed strong resistance.

**Figure 6:**
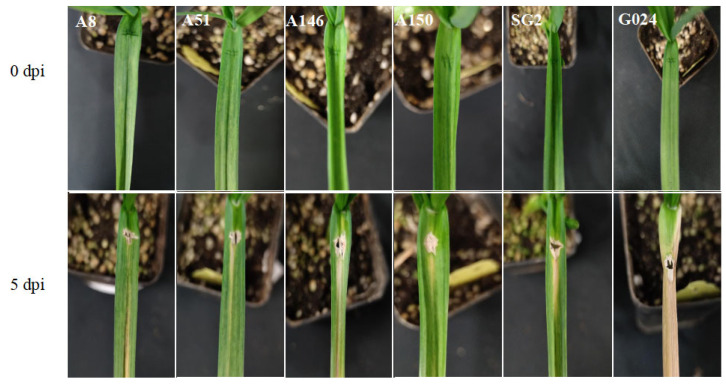
The symptoms were observed after 0, 5 days of inoculation with *Pleospora herbarum*.

**Figure 7:**
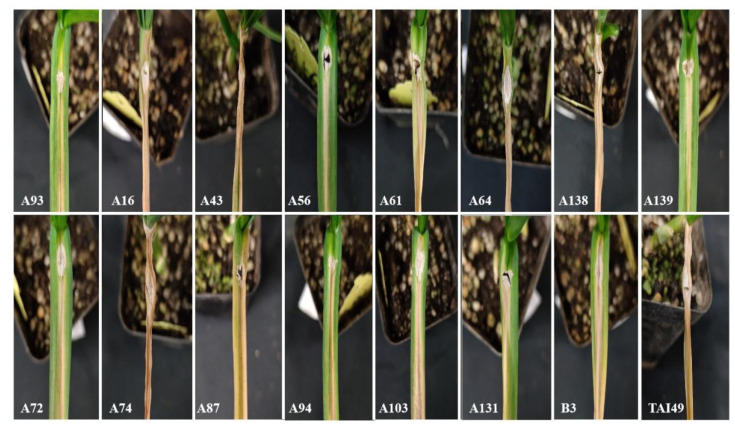
*Pleospora herbarum* was inoculated and symptoms were observed after 5 days.

The 21 mutant lines and the G024 cultivar were inoculated in the greenhouse to further identify and screen resistant lines. At 5 days post-inoculation (dpi), the degree of leaf blight infection on the leaves differed between the 21 mutant lines and G024 (Figure 6 and Figure 7). Almost the whole leaf of the G024 control was infected. Among the 21 mutant lines, the lesion area in 5 mutant lines (A8, A51, A146, A150, SG2) was significantly smaller than that in G024 (Figure 6), and the lesions in 10 mutant lines (A16, A43, A61, A64, A138, A74, A87, A103, B3, TAI49) spread to the whole leaf and reached the state of necrosis (Figure 7).

### 2.5. Differentially Expressed Genes between A150 and G024 after Pleospora herbarum Infection

**Figure 8:**
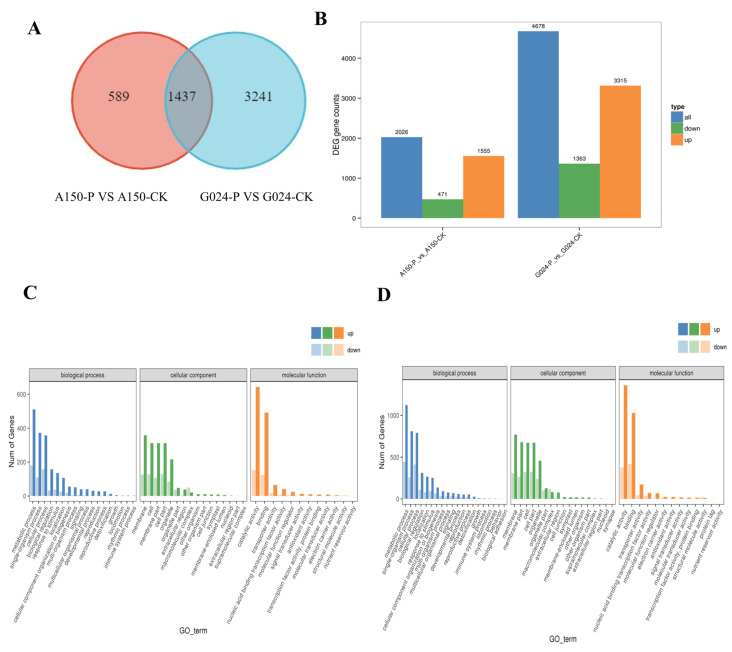
RNA-seq analysis between A150 and G024 after inoculation with *Pleospora herbarum*. (**A**) Number of differentially expressed genes at 48 h post-inoculation (hpi). (**B**) Number of up- and downregulated DEGs at 48 hpi. (**C**,**D**) Classification cartogram of GO annotation of DEGs.

A total of 2026 DEGs were identified between A150 and its control, while 4678 DEGs were identified between G024 and its control (Figure 8A). Among those in G024, 3315 DEGs were significantly upregulated and 1363 DEGs were significantly downregulated. In the resistant A150 genotype, 1555 DEGs were significantly upregulated and 471 DEGs were significantly downregulated (Figure 8B). After *Pleospora herbarum* infection, it was noted that there were more upregulated than downregulated DEGs in both genotypes, and more DEGs were identified in the G024 genotype than in the A150 genotype. Multiple genes were found to be involved in the interaction between garlic and *Pleospora herbarum*.

### 2.6. Differentially Expressed Genes Are Distributed in Different Functional Pathways

Gene Ontology (GO) term enrichment was used to investigate the functions of the DEGs between A150 and G024. In the cellular component category, the DEGs of A150 were significantly enriched in the membrane (GO:0016020), cell (GO:0005623), membrane part (GO:0044425), and cell part (GO:0044464). In the molecular function category, the DEGs of A150 were significantly enriched in catalytic activity (GO:0003824) and binding (GO:0005488). In the biological process category, the DEGs of A150 were significantly enriched in metabolic process (GO:0008152), single-organism process (GO:0044699), and cellular process (GO:0009987). Also, it was noted that there were more upregulated than downregulated DEGs in the most significantly enriched terms (Figure 8C). The results of G024 were similar to those of A150, but in the most significant enrichment terms, there were more DEGs of G024 than A150 (Figure 8D).

**Figure 9:**
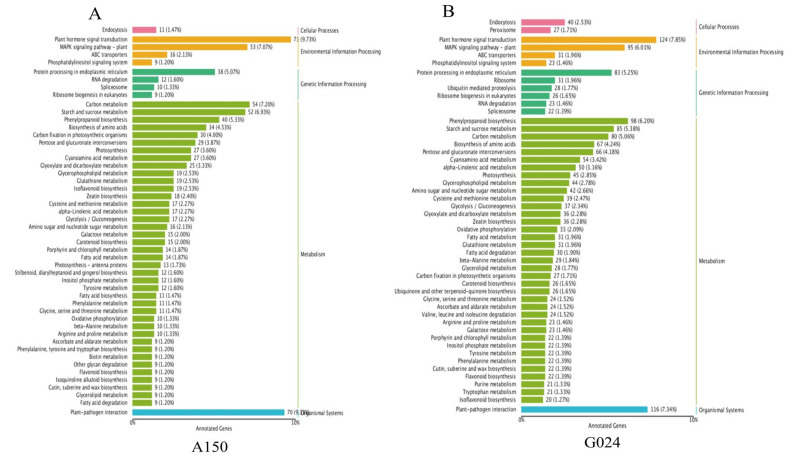
RNA-seq analysis of metabolite pathways in (**A**) A150 and (**B**) G024 under *Pleospora herbarum* infection.

Based on the KEGG descriptions of the entire set of DEGs, the number of genes with particular annotation terms was significantly overrepresented (Figure 9A,B). There were 112 biochemical pathways identified in A150 compared to the control, among which 73 DEGs were mainly enriched in hormone signal transduction, including SA, JA, and ET. While 70 important DEGs were identified in plant defense responses during plant–pathogen interaction, some other DEGs were found to be enriched in the MAPK signaling pathway, starch and sucrose metabolism, and phenylpropanoid biosynthesis (Figure 9A). More DEGs were identified in the susceptible G024 strain, in which 124 DEGs were involved in plant hormone signal transduction, 116 DEGs in plant–pathogen interaction, 98 DEGs in the phenylpropanoid biosynthesis pathway, 95 DEGs in the MAPK signaling pathway, and 85 in starch and sucrose metabolism (Figure 8B).

**Figure 10:**
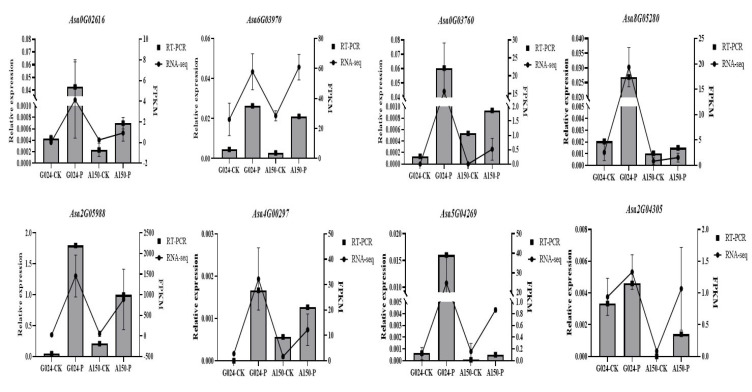
RNA-seq results were validated using qRT–PCR.

To verify the accuracy of the RNA-seq data in this study, eight DEGs (Asa6G03970, Asa0G02616, Asa0G03760, Asa8G05280, Asa2G05988, Asa4G00297, Asa5G04269, Asa2G04305) related to the MAPK signaling pathway were selected to measure their relative transcription via qRT-PCR analysis. The results of qRT-PCR for the eight selected genes were consistent with the RNA-seq data, suggesting that the DEGs in the RNA-seq analysis were reliable (Figure 10).

## 3. Discussion

It is difficult to improve the main agronomic traits and stress resistance of garlic by traditional genetic breeding, mainly because garlic undergoes vegetative propagation. Therefore, mutation breeding of garlic cloves has been considered to be an effective way to cultivate new varieties or improved lines based on elite varieties. The frequency of mutagenesis in the progeny can be increased to about 11%, 100 times the natural (background) frequency [21]. In this study, EMS chemical mutagen was used for mutagenesis of garlic bulbils, greatly improving the overall mutagenesis efficiency of garlic.

EMS is widely used for chemical mutagenesis and is currently recognized as having obvious and stable effects. EMS-induced point mutations or chromosomal deletions in seeds and callus can be stably passed on to offspring. A good mutagenic effect can only be obtained when the mutagen dose reaches the optimum concentration. The induction effect of EMS is not only related to the mutagen concentration, but also the treatment time [22]. When the concentration of EMS is increased or the treatment time is prolonged, the mutation rate of the material will increase and the germination rate will decrease [22,23], because EMS causes physiological damage to the mutagenic material, and this damage will also increase with increased EMS concentration or treatment time. Therefore, in order to obtain the best mutagenic effect, it is necessary to determine the experimental conditions where the mutation rate will reach a certain level, and it should not lead to excessive plant death. Generally, the combination of EMS concentration and treatment time that leads lethality of half the plants is considered to be the best treatment. In this experiment, the optimal mutagenic combination for garlic bulbils was 1.0% EMS for 8 h.

EMS mutagenesis produces many mutations with adverse agronomic traits. It was observed in this experiment that the mutation rate of phenotypic traits in the field population of M_1_ mutants was about 6.2%, and the mutants were screened for a wide range of mutations, including plant height, leaf color, pseudo-stem, bulb, and other aspects, such as leaf albinism, leaf deformity, bulb deformity, plant dwarfism, and involucre enlargement. Phenotypic mutations were also observed in other studies. Xin et al. (2008) [24] treated sorghum Tx623 with different concentrations of EMS to obtain mutants with different dwarfing, leaf color, and leaf shape. Furthermore, EMS showed very high efficiency in tomatoes by inducing different morphological and functional mutations [25]. In addition, seedlings with lethal albinism were found in a study by Alcantara et al. (1996) [22].

At present, the methods for identifying resistance to garlic leaf blight are not perfect. Cheng et al. (2010) [26] used in vitro inoculation and identification to identify disease resistance, and the results were consistent with the field phenotype of disease resistance. Therefore, in this experiment, 21 disease-resistant mutant lines were screened by natural infection in the field. However, natural infection in the field is easily affected by the environment. Therefore, it is necessary to further identify disease resistance by performing live inoculation in the greenhouse. We used the wound inoculation method to identify the disease resistance of leaves, and ensured that the inoculum dose, wound size, and position of control and disease-resistant mutants were exactly the same to ensure that they would not affect the results. From the results of live inoculation, it can be seen that the degree of leaf infection was significantly different between G024 and the 21 disease-resistant mutants at 5 dpi (Figure 6 and Figure 7). It is clear from Figure S1 that there was little difference between G024 and the mutants at 1 dpi. At 3 dpi, the leaves of G024 were seriously infected, while A150 and A51 only had lesions that did not spread, and the leaves of SG2 showed a slight trend of spreading lesions. At 7 dpi, the leaves of G024 reached the state of necrosis, while lesions on the leaves of A150 and A51 began to spread. This indicates that it is feasible to use EMS mutagenesis technology to create resistant varieties of garlic. Our results are consistent with those of previous studies reporting that EMS mutagenesis increased disease resistance in some plants [27,28]. In addition, it can be seen from Supplementary Table S1 that the morphological traits of G024 and three mutants were also different; among them, every morphological indicator of SG2 was better compared to G024. Therefore, it is expected that using EMS mutagenesis technology to breed varieties with disease resistance and good growth traits will be effective.

Garlic varieties A150 and G024 were selected for RNA sequencing. Transcriptome analysis was carried out after *Pleospora herbarum* infection in A150 and G024, and the results revealed 2026 and 4678 differentially expressed genes (DEGs), respectively. These DEGs were highly enriched in hormone signaling pathways, plant–pathogen interactions, and the MAPK signaling pathway.

## 4. Materials and Methods

### 4.1. Plant Material

The G024 garlic variety used in this experiment was provided by the Garlic Research Group, College of Horticulture, Northwest A&F University, Shaanxi, China. The results of inoculation showed that this strain has low resistance to leaf blight.

### 4.2. Establishment of EMS Mutation Technology

G024 garlic cloves were subjected to mutagenesis via EMS. We set up a crossed factorial experiment using a series of mutagen concentrations and immersion times. Garlic cloves were incubated for 4, 8, 12, 16, and 24 h in 0, 1.0, and 1.5% mutagen, for a total of 11 treatment combinations. After the treatment, the garlic cloves were rinsed 1 time with 1 M Na₂S₂O₃ and 3 times with 0.1 M Na₂S₂O₃, and then soaked in distilled water for 30 min. Thereafter, the treated garlic cloves were planted in 32-hole trays, and there were 3 replications with 50 garlic cloves per replicate. The growth and germination of plants were recorded, and the germination rate (GR) was calculated (no germination or the presence of yellow leaves was identified as no germination on the 28th day).

Germination rate (GR) = (Number of germinated garlic cloves/Total number of garlic cloves) × 100%

### 4.3. Field Experiments and Plant Growth

A total of 5000 garlic cloves (G024) were mutated in the optimal EMS mutagenesis conditions (1% EMS for 8 h). Garlic cloves of the M_0_ generation treated by mutagenesis were seeded at the Yangling Experimental Demonstration Station of Northwest A&F University in September 2017. Garlic G024 that was not treated with EMS was used as a control.

In April 2018, field phenotypes of 5000 M_1_ mutants were examined and identified. Morphological traits of seedlings, leaves, and stems were observed, and obvious morphological mutations were labeled and photographed. The garlic lines with good performance in the field were selected for sowing the following year. In the third generation, 169 lines remained. The expression and isolation of agronomic traits of 169 M_2_ and M_3_ mutants were analyzed, including leaf length (LL), leaf width (LW), leaf number (LN), pseudo-stem height (HP), stem diameter (SD), garlic weight (GW), garlic diameter (GD), garlic height (GH), and petal number (PN).

### 4.4. Screening and Identification of EMS Mutated Lines Resistant to Leaf Blight

#### 4.4.1. Phenotypic Observations

In order to screen mutant lines resistant to leaf blight, data were recorded starting from the planting date using the field natural identification method [29]. For the field identification of trial varieties (mutants and G024), no fungicide was used for the whole year. Ten plants from each line were randomly selected to investigate disease severity and calculate the disease index. Leaf blight incidence was scored by determining the percentage of infection on garlic leaves using a 0–9 scale (0: no infection; 1: spots accounted for less than 5% of total leaf area; 3: diseased spot areas accounted for 6–25% of total leaf area; 5: diseased spot areas accounted for 26–50% of total leaf area; 7: diseased spot areas accounted for 51–75% of total leaf area; 9: diseased spot areas accounted for more than 76% of total leaf area) [30].

Disease index = (Sum of individual ratings × 100%)/(10 × 9)

#### 4.4.2. Fungal Inoculation

Isolated and purified garlic leaf blight fungus (*Pleospora herbarum*) was used for inoculation in the greenhouse. Fungal plugs 0.5 cm in diameter were taken and used to culture fungal mycelia on potato dextrose agar (PDA) plates. After the mycelium had covered the entire medium, the 1.5 cm diameter plugs with actively growing fungus were used to inoculate mung bean broth, followed by shaking at 200 rpm at 28 °C for 24 h for macroconidia production. The mung bean broth was prepared by steeping 20 g of dry mung beans in 500 mL of boiling distilled water for 20 min. The extract was autoclaved and cooled overnight before inoculating the PDA plugs with the fungal mycelia [10]. After 24 h, the macroconidia concentration was measured using a hemacytometer, and a suspension at a concentration of 1 × 10^6^ spores/mL was used for inoculation [31].

Garlic G024 was inoculated and screened for identification of resistant mutants. A sterilized scalpel was used to cut a number sign (#) shaped wound 2 cm near the pseudo-stem, and 10 μL of spore suspension was dropped on each wound. Ten seedlings of each line were selected for inoculation. After inoculation, the plants were grown in a controlled growth chamber with temperature maintained at 28 °C, with a 16 h/8 h light/dark photoperiod and 80–90% relative humidity. The disease symptoms were investigated at 0, 1, 3, 5, and 7 d after inoculation, and the resistant strains were further screened.

### 4.5. Transcriptome Analysis and Functional Annotation

Garlic varieties A150 and G024 were selected for RNA sequencing. Leaf samples were collected at 48 h post-inoculation (hpi) and immediately frozen in liquid nitrogen and stored at −80 °C. Three seedlings were used as one repetition, and there was a total of three repetitions. The samples were sent to Beijing Biomarker (www.biocloud.net) for RNA sequencing on 27 September 2022. The reference genome for transcriptome analysis was obtained from https://figshare.com/articles/dataset/garlic_genome_and_transcriptome_resources/12570947/1 (accessed on 30 May 2023). 

In order to determine the significance of DEGs in the RNA-seq results, we set the significance threshold to |Log2 (fold change)| ≥ 1.0, and the adjusted *p* value was ≤0.01. The level of gene expression was based on the number of expected fragments per kilobase of transcript per million mapped reads (FPKM).

### 4.6. Validation of RNA-Seq Data

To confirm the accuracy and reproducibility of the Illumina RNA-Seq results, 8 candidate DEGs were selected for testing using qRT-PCR. The leaves of A150 and G024 plants were sampled at 48 hpi, frozen immediately in liquid nitrogen, and stored at −80 °C. Total RNA was extracted using a Biospin Plant Total RNA Extraction Kit (DiNing, Beijing, China). The cDNA fragments were synthesized using a Transcriptor First Strand cDNA Synthesis Kit (Roche, Mannheim, Germany). All qRT-PCR tests were run with SYBR Green (GeneStar, Beijing, China) using a QuantStudio^®^5 real-time PCR machine (Life Technologies, Gaithersburg, MD, USA). Briefly, 10 μL of mix were prepared containing 1 μL each of forward and reverse gene-specific primers (Supplementary Table S1), 3 μL of cDNA, and 5 mL of RNase-free water. The process for PCR amplification was as follows: initial denaturation at 94 °C for 15 min, followed by 40 cycles at 94 °C for 15 s, then 60 °C for 30 s. UBQ [32] was used as reference gene to normalize the transcript levels for each sample, and the final data were calculated using the formula 2^−ΔΔCT^.

### 4.7. Data Collection and Statistical Analysis

All data were analyzed using Excel 2010 and SPSS 20 (IBM, Chicago, IL, USA) for Windows PC. The 9 plant traits of G024 and the mutants were used as input values for principal component analysis (PCA) to check for similarities and differences in agronomic traits among the samples; this was performed using GraphPad Prism 9.0.0.121.

## 5. Conclusions

In this study, we created a garlic mutant library by performing EMS mutagenesis of the garlic G024 cultivar. By investigating the agronomic traits of the mutants, we found that, compared with the control G024 cultivar, many traits of the mutants were obviously improved, indicating that the agronomic traits of the plant could be improved by EMS mutagenesis. In addition, the disease index of M_3_ mutants naturally infected with leaf blight in the field was investigated, and 21 mutant lines with leaf blight resistance were screened out. Finally, the 21 screened mutant lines underwent live inoculation in the greenhouse, and 3 mutants with excellent disease resistance were further screened. Taken together, these results lay a foundation for the further creation of new garlic germplasm resources.

## Figures and Tables

**Table 1:** Garlic clove germination after treatment with EMS.

EMS Concentration (%)	EMS Treatment Time (h)	No. of Cloves	No. of Explants for Germination	Germination Rate (%)
0%	0	50	49.6 ± 0.33	99.33 ± 1.15 a
1.00%	4	50	35 ± 0.57	70.00 ± 2.00 b
	8	50	24.6 ± 0.88	49.33 ± 3.05 c
	12	50	16.6 ± 0.88	32.66 ± 3.05 e
	16	50	10 ± 1.15	20.00 ± 4.00 f
	24	50	3 ± 1.00	6.00 ± 3.46 h
1.50%	4	50	26.6 ± 1.20	51.33 ± 4.16 c
	8	50	21.6 ± 1.45	43.33 ± 5.03 d
	12	50	11.3 ± 0.88	22.66 ± 3.05 f
	16	50	6.3 ± 0.88	12.66 ± 3.05 g
	24	50	0.6 ± 0.33	1.33 ± 1.15 h

Statistical significance was evaluated by two-way ANOVA. In last column, different lowercase letters represent significantly different mean values.

**Table 2:** Phenotypic traits of M_1_ mutants.

Mutant Organ	Mutation Characteristics	Number of M_1_ Mutations	M_1_ Mutation Frequency
Blade	Albinism	1	0.29‰
	Mosaicism	3	0.88‰
	Curly leaves	2	0.58‰
Involucre	Expansion	6	1.7‰
Tiller	Tiller abnormality	3	0.88‰
Plant	Dwarfism	1	0.29‰
	Robustness	161	4.80%
	Disease resistance	17	0.53%
Total		199	6.20%

**Table 3:** Comparison of morphological traits between M2 mutants and G024.

Trait	G024	Mean of Mutants	Range	CV
Leaf length (cm)	43.53 ± 0.13	54.18 ± 5.4	43.53–66.33	0.12
Leaf width (mm)	24.38 ± 0.17	29.68 ± 3.98	21.44–36	0.11
Pseudo-stem height (cm)	37.17 ± 0.15	36.62 ± 2.82	27.67–43.33	0.01
Stem diameter (mm)	13.1 ± 0.22	16.31 ± 5.19	11.67–21.46	0.12
Leaf number	7.7 ± 0.12	7.85 ± 0.86	6.67–9.33	0.01
Garlic weight (g)	23.1 ± 0.12	34.78 ± 4.65	13.97–55.19	0.12
Garlic height (mm)	29.77 ± 0.02	33.23 ± 2.66	24.04–39.85	0.06
Garlic diameter (mm)	40.09 ± 0.09	47.23 ± 3.19	32.63–56.53	0.09
Petal number	10 ± 0.1	12.77 ± 8.22	7–17.67	0.13

**Table 4:** Comparison of morphological traits between M_3_ mutants and G024.

Trait	G024	Mean of Mutants	Range	CV
Leaf length (cm)	42.67 ± 1.20	54.80 ± 0.29	41.67–63.67	0.14
Leaf width (mm)	27.17 ± 1.08	25.25 ± 0.14	21.25–34.34	0.04
Pseudo-stem height (cm)	42.33 ± 1.67	46.12 ± 0.28	36.67–56.67	0.05
Stem diameter (mm)	15.93 ± 1.01	13.64 ± 0.11	11.06–18.05	0.08
Leaf number	6.67 ± 0.33	6.83 ± 0.03	6.00–8.33	0.01
Garlic weight (g)	29.41 ± 1.87	48.50 ± 0.59	18.83–63.22	0.27
Garlic height (mm)	31.53 ± 0.82	33.08 ± 0.16	25.82–38.12	0.03
Garlic diameter (mm)	44.81 ± 1.76	48.50 ± 0.26	39.30–56.00	0.04
Petal number	12.33 ± 0.33	12.47 ± 0.09	9.67–15.67	0.006

## Data Availability

The original contributions presented in the study are included in the article or the Supplementary Materials. Further inquiries can be directed to the corresponding author.

## Supplementary Materials

The following supporting information can be downloaded at: https://www.mdpi.com/article/10.3390/ijms241411819/s1.
