# Supplementary material for: Screening and Identification of Garlic Leaf Blight (Pleospora herbarum)-Resistant Mutants Induced by Ethyl Methane Sulphonate
Source: Int J Mol Sci. 2023 Jul 23;24(14):11819. doi: 10.3390/ijms241411819 (PMC10380478; doi:10.3390/ijms241411819)
Supplement: Supplementary file 1 [file ijms-24-11819-s001.zip › ijms-2454568-supplementary.pdf]

Supplementary

Supplementary Table S1. Comparison of morphological traits between three mutants and G024.

Traits	G024	A150	SG-2	A51
Leaf length(cm)	43.64	40.7	58.74	49
Leaf width(mm)	28.82	24.44	31.4	25.4
height of pseudo-stem(cm)	20.4	24.38	29.8	26.92
Stem diameter(mm)	15.06	13.88	16.89	14.59
Leaf number	7.6	7.6	8.6	8.2
Garlic weight(g)	20.28	26.41	36.8	15.83
Garlic height	31.39	34.79	35.1	31.07
Garlic diameter(mm)	39.75	42.45	49.59	38.83
Petal number	10.6	9.2	11.8	8.8

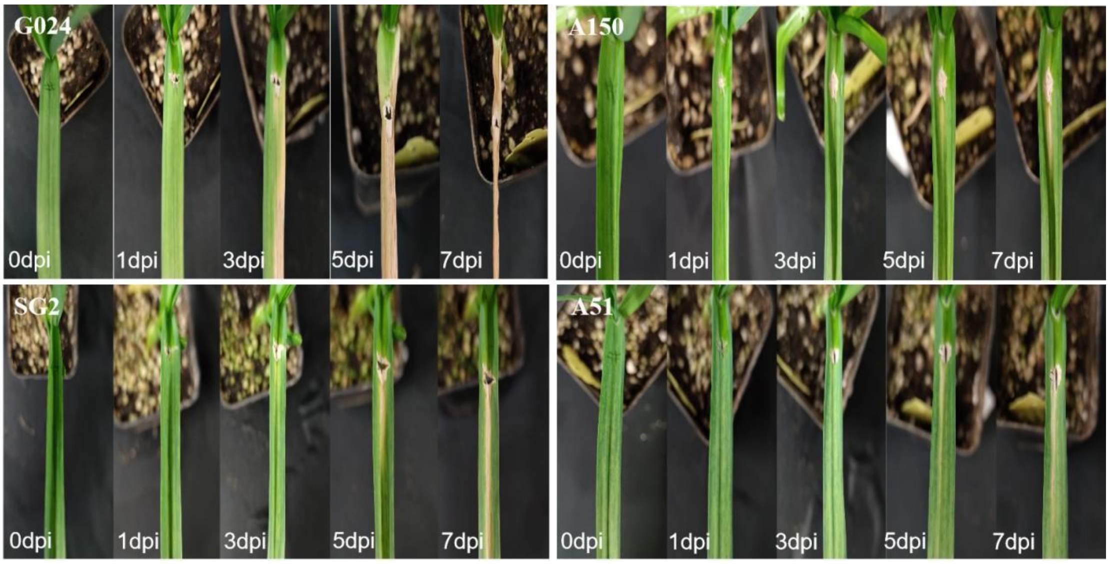

Figure S1. Disease symptoms observed at 0, 1, 3, 5, 7 days post-inoculation(dpi).
